# Inhibition of Gap Junctional Intercellular Communication Upregulates Pluripotency Gene Expression in Endogenous Pluripotent Muse Cells

**DOI:** 10.3390/cells11172701

**Published:** 2022-08-30

**Authors:** Khaled Hatabi, Yukari Hirohara, Yoshihiro Kushida, Yasumasa Kuroda, Shohei Wakao, James Trosko, Mari Dezawa

**Affiliations:** 1 Department of Stem Cell Biology and Histology, Tohoku University Graduate School of Medicine, Sendai 980–8575, Japan; 2 Regenerative Medicine Division, Life Science Institute, Inc., Tokyo 135-0004, Japan; 3 Department of Pediatrics and Human Development, College of Human Medicine, Michigan State University, East Lansing, MI 48824, USA

**Keywords:** muse cells, pluripotency, gap junction inhibition, Cx43, YAP

## Abstract

Gap junctions (GJ) are suggested to support stem cell differentiation. The Muse cells that are applied in clinical trials are non-tumorigenic pluripotent-like endogenous stem cells, can be collected as stage-specific embryonic antigen 3 (SSEA-3+) positive cells from multiple tissues, and show triploblastic differentiation and self-renewability at a single cell level. They were reported to up-regulate pluripotency gene expression in suspension. We examined how GJ inhibition affected pluripotency gene expression in adherent cultured-Muse cells. Muse cells, mainly expressing gap junction alpha-1 protein (*GJA1*), reduced GJ intercellular communication from ~85% to 5–8% after 24 h incubation with 120 μM 18α-glycyrrhetinic acid, 400 nM 12-O-tetradecanoylphorbol-13-acetate, and 90 μM dichlorodiphenyltrichloroethane, as confirmed by a dye-transfer assay. Following inhibition, *NANOG*, *OCT3/4*, and *SOX2* were up-regulated 2–4.5 times more; other pluripotency-related genes, such as *KLF4*, *CBX7*, and *SPRY2* were elevated; lineage-specific differentiation-related genes were down-regulated in quantitative-PCR and RNA-sequencing. Connexin43-siRNA introduction also confirmed the up-regulation of *NANOG*, *OCT3/4*, and *SOX2*. YAP, a co-transcriptional factor in the Hippo signaling pathway that regulates pluripotency gene expression, co-localized with GJA1 (also known as Cx43) in the cell membrane and was translocated to the nucleus after GJ inhibition. Adherent culture is usually more suitable for the stable expansion of cells than is a suspension culture. GJ inhibition is suggested to be a simple method to up-regulate pluripotency in an adherent culture that involves a Cx43-YAP axis in pluripotent stem cells, such as Muse cells.

## 1. Introduction

The cells of multicellular organisms have many different ways to communicate with neighboring cells to maintain cellular and tissue homeostasis. Gap junction (GJ) channels are one of the representative systems that enables the exchange of small molecules, second messengers, and electrical signals to connect the cytoplasmic space of two adjacent cells. GJs consist of connexin (Cx) proteins that oligomerize into hexamers to form hemi-channels, namely connexons. One connexon is presented from each cell to form a GJ channel between the two cells [1,2].

GJ intercellular communication (GJIC) is also suggested to be important in regulating stem cell differentiation. For example, the ablation of GJIC led to the disruption of differentiation in embryoid bodies derived from mouse embryonic stem (ES) cells [3]. Moreover, human induced pluripotent stem cells (iPSCs) programming process is accompanied by GJIC disruption, which is re-established again after their differentiation into somatic cells [4].

Multilineage-differentiating stress-enduring (Muse) cells exhibit pluripotent-like characteristics. They are endogenous stem cells found as pluripotency surface marker stage-specific embryonic antigen 3 (SSEA-3)-positive in the bone marrow, peripheral blood, and the connective tissues of various organs, which exhibit stress tolerance by having a higher capacity for sensing and repairing DNA damage when compared to other stem cells, such as mesenchymal stem cells (MSCs) [5,6,7,8,9,10]. They, also, occupy approximately 1–5% of the total population of cultured MSCs and fibroblasts [6]. Muse cells express pluripotency markers, such as POU class 5 homeobox 1 (*POU5F1*) (also known as *OCT3/4*), *NANOG*, and SRY (sex determining region Y)-box 2 (*SOX2*), at moderate levels when compared with those in ES and iPSCs, and can differentiate, spontaneously, not only into adipogenic-, chondrogenic-, and osteogenic-lineage cells, but also into other mesodermal-lineage cells (such as cells positive for homeobox protein NKx2.5, desmin, and alpha-smooth muscle actin), as well as into endodermal- (GATA binding factor 6, cytokeratin 7, and alpha-fetoprotein) and ectodermal (microtubule-associated protein 2 and neurofilament)-lineage cells from a single cell in vitro without cytokine induction [6,11,12]. The triploblastic differentiation from a single Muse cell is reproducible over generations, and thus, they were suggested to be self-renewable [6,11]. Not only does this occur spontaneously, but they also differentiate in vitro at a high rate (~80–95%) as a result of cytokine inductions into triploblastic lineage cells such as: cardiac-, hepatic-, and neural-lineage cells, as well as adipocytes, osteocytes, keratinocytes, and melanocytes [11,13,14,15,16]. They also differentiated, spontaneously, into mesodermal-, ectodermal- and endodermal-lineage cells in vivo in animal disease models, for example, differentiation into cardiomyocytes and vascular cells was reported in rabbit acute myocardial infarction model; into neuronal and glial cells was reported in mouse and rat stroke models; into podocytes, mesangial cells and endothelial cells was reported in mouse chronic kidney disease model; into hepatocytes, sinusoidal cells and Kupffer cells was reported in mouse liver damage model [12,17,18,19,20].

Circulating endogenous Muse cells and intravenously administered exogenous Muse cells are able, selectively, to home to the damaged tissues by sensing sphingosine-1-phosphate (S1P), one of the damage signals produced by damaged cells, and replace the damaged/apoptotic cells by spontaneous differentiation into the corresponding cell types that comprise the tissue after homing and repair the tissue [17,18,19,20]. Since they have an immune privilege, that may be partly explained by the expression of a human leukocyte antigen (HLA)-G (that is relevant to immunosuppression in the placenta), intravenously injected donor-derived Muse cells escape from immune rejection and survive as functional cells in the host tissue for over half a year without using immunosuppressants [20]. Currently, clinical trials of stroke, acute myocardial infarction, epidermolysis bullosa, and spinal cord injuries are being conducted through the intravenous injection of a human clinical-grade Muse cell-based formulation (CL2020) without HLA-matching or an immunosuppressant, under the permission of the regulatory authorities. The safety and effectiveness of CL2020 is reported in studies of acute myocardial infarction and epidermolysis bullosa [21,22].

It has been recently shown that the expression of gap junction alpha-1 protein (*GJA1*), also known as connexin 43 (Cx43), is remarkably different between the two states of pluripotency, the naïve state and the primed state. Pluripotent stem cells are classified into two discrete states, ‘naïve’ and ‘primed’, based on multiple functional differences, including their growth factor dependency, intracellular signaling, marker expression, and differentiation potential; human primed pluripotent stem cells up-regulate Cx43 expression in comparison to those of the naïve state [23]. Furthermore, naïve pluripotent stem cells seem to be less affected by the pharmacological ablation of GJIC when compared to primed pluripotent stem cells [23]. Interestingly, Muse cells are more similar to primed pluripotent stem cells than to naïve pluripotent stem cells for their dependency on the basic fibroblast growth factor (bFGF), rather than the leukemia inhibitory factor (LIF), to maintain their proliferation and self-renewability [5].

Here, we show that GJIC inhibition in Muse cells with GJ inhibitors, such as dichlorodiphenyltrichloroethane (DDT), 12-O-tetradecanoylphorbol-13-acetate (TPA), and 18α-glycyrrhetinic acid (18α-GA), as well as the introduction of siRNA for *GJA1*, up-regulates the expression of pluripotency-related markers in the adherent culture. An RNA-seq analysis suggested that GJIC inhibition suppressed the expression of cell differentiation-related pathways. Finally, our data suggested that there is involvement from the Cx43/Yes-associated protein (YAP) in the pluripotency gene up-regulation in GJ-inhibited Muse cells. YAP, a key transcriptional factor in the Hippo signaling pathway that plays an important role in cell proliferation, stem cell self-renewal, and tissue formation, is suggested to be anchored to connexins in the cell membrane and is released from the connexins and translocated to the nucleus under the presence of the GJ inhibitors. Since YAP is known to bind to the promoter regions of pluripotency genes, translocated YAP might have up-regulated the pluripotency genes.

Muse cells, as well as ES cells and iPSCs, were shown to up-regulate pluripotency gene expression when they were cultured in suspension [7,13,24,25]. Adherent culture is, however, more suitable for the stable expansion of cells on large scale than a suspension culture is. GJIC inhibition might be a simple method that enables both the stable cell expansion in adherent culture and the up-regulation of pluripotency gene expression.

## 2. Materials and Methods

### 2.1. Cell Culture and Isolation of Muse Cells

Human bone marrow-derived mesenchymal stem cells (hMSCs) were purchased from Lonza (Basel, Switzerland). Cells were cultured in Minimum Essential Medium Eagle (αMEM) (Sigma-aldrich, St. Louis, MO, USA) with 10% fetal bovine serum (FBS) (Hyclone, Logan, UT, USA), 1 ng/mL basic fibroblast growth factors (bFGF) (Miltenyi Biotec, Bergisch Gladbach, Germany), and 1× kanamycin (Gibco, Waltham, MA, USA) in a humidified incubator adjusted to 5% CO_2_ and 37 °C.

hMSCs (passage 8) at 100% confluency were subjected to cell sorting to isolate Muse cells that were collected as cells positive for SSEA-3 and non-Muse cells that are negative for SSEA-3, as reported previously [6]. In brief, hMSCs were incubated with anti-SSEA-3 rat antibody (1:1000, Biolegend, San Diego, CA, USA), followed by anti-rat IgM antibody conjugated with FITC (1:100, Jackson ImmunoResearch, West Grove, PA, USA). Sorting gates were appropriately set to collect SSEA-3-positive and SSEA-3-negative cell populations. All sorting was performed using BD FACS Aria II SORP Flow Cytometer Cell Sorter (Becton Dickinson, Franklin Lakes, NJ, USA).

Human cardiomyocyte cell line (AC-16) cells were purchased from Millipore (Burlington, MA, USA), and were cultured in Dulbecco’s Modified Eagle Medium/Nutrient Mixture F-12 (Gibco), containing 12.5% fetal bovine serum (Hyclone) and 1× kanamycin (Gibco).

### 2.2. Reagents Preparation

18α-GA was purchased from Sigma (#G8503), and a 25 mM stock solution was prepared by dissolving in DMSO (Wako, Osaka, Japan) and was preserved at −30 °C. A fresh working solution of 120 μM was used for 24 h incubation in the main experiment.

TPA was purchased from AdipoGen Life Sciences (Liestal, Switzerland, #AG-CN2-0010), and a 500 μM stock solution was prepared by dissolving in DMSO and was preserved at −30 °C. A fresh working solution of 400 nM was used for 24 h incubation in the main experiment.

DDT was purchased from Tokyo Chemical Industry (TCI, Tokyo, Japan, #T0379), and a 250 mM stock solution was prepared by dissolving in DMSO and was preserved at −30 °C. A fresh working solution of 90 μM was used for 24 h incubation in the main experiment.

Etoposide was purchased from Sigma (#E1383), and a 50 mM stock solution was prepared by dissolving in DMSO and was preserved at −30 °C.

### 2.3. Quantitative PCR (qPCR)

Total RNA was isolated using RNA Isolation Kit (NucleoSpin RNA XS, Takara Bio, Shiga, Japan), according to the manufacturer’s instructions. Total RNA concentration and quality were confirmed by using Nanodrop 1000 spectrophotometer (Thermo Fisher Scientific, Waltham, MA, USA). cDNA synthesis was performed by reverse-transcription (RT), using SuperScript III first-strand synthesis system (Thermo Fisher Scientific), according to the manufacturer’s protocol, and Takara Thermal PCR Cycler (Takara Bio). TaqMan primers used for qPCR were: Hs00748445_s1 for *GJA1* (Cx43), Hs00979198_m1 for *GJA5* (Cx40), Hs00271416_s1 for *GJC1* (Cx45), Hs00252713_s1 for *GJC2* (Cx47), Hs00987388_s1 for *GJD3* (Cx31.9/Cx30.2), Hs00542133_m1 for *GJD4* (Cx40.1), Hs04399610g1 for *NANOG*, Hs04260367gH for *OCT3/4*, Hs01053049s1 for *SOX2*, and Hs03023943g1 for *ACTB*.

qPCR was performed using 7500 Fast Real-Time PCR System (Applied Biosciences, Waltham, MA, USA). β-actin was used as endogenous control. qPCR data analysis was performed, using (2^−ΔΔCT^) relative quantification method in all calculations.

### 2.4. Western Blot

Cells were first washed with cold 1× phosphate buffered saline (PBS) three times, collected into 1 mL cold 1× PBS by using cell scrapper, and transferred into 1.5 mL microcentrifuge tubes. Cell pellets were obtained by centrifuging for 400× *g* for 5 min and were lysed using RIPA lysis buffer (Thermo Fisher Scientific) with Protease Inhibitor Cocktail (Thermo Fisher Scientific). Next, the cell lysate was centrifuged for 10,000 rpm for 10 min, and then the supernatant was transferred to a new tube for Western blotting analysis. Alternatively, the Cell Fractionation Kit (Cell Signaling, #9038, Danvers, MA, USA) was used to obtain nuclear and cytoplasmic fractions, according to the manufacturer’s instructions. Protein quantification was done using BCA Protein Assay Kit (Thermo Fisher Scientific). SDS-PAGE was carried out by 10% SDS-PAGE gels (Fujifilm, Tokyo, Japan) and then proteins were transferred to PVDF membranes (Millipore). Membranes were blocked by 5% skim milk for 1 h (Nacalai Tesque, Kyoto, Japan) in room temperature and were then incubated with the following primary antibodies overnight at 4 °C: anti-GJA1 (Cx43) antibody (1:2000, Abcam #ab11370, Cambridge, UK), anti-GJC1 (Cx45) antibody (1:1000, Abcam #ab78408), anti-YAP antibody (1:1000, Cell Signaling #14074), anti-Histone H3 antibody (1:2000, Cell Signaling #9715), anti-alpha Tubulin antibody (1:5000, Abcam #ab7291), and anti-beta Actin antibody (1:10,000, Abcam #ab6276). The following secondary antibodies were used to detect the target antibody signal for 1 h at room temperature: anti-rabbit IgG goat antibody conjugated to HRP (1:10,000, Jackson ImmunoResearch) and anti-mouse IgG goat antibody conjugated to HRP (1:10,000, Jackson ImmunoResearch). Skim milk (5%) was used for primary and secondary antibody dilution. Finally, the signal was measured using Luminescent image analyzer LAS-4000mini (Fujifilm), and the intensity of each signal band was analyzed using ImageJ (https://imagej.nih.gov/ accessed on 23 June 2019).

### 2.5. Immunofluorescent Imaging

Cells were first washed with cold 1× PBS twice and then fixed using 4% paraformaldehyde (PFA) (Nacalai Tesque) for 15 min at room temperature. Then, cells were incubated with Blocking solution (20% Blockace powder (KAC Co., Ltd., Hyogo, Japan)/5% BSA (Nacalai Tesque)/0.3% Triton X-100 (Wako)/1× PBS (Nacalai Tesque) for 30 min at room temperature for blocking, and were subsequently incubated with primary antibody overnight at 4 °C. Primary antibodies used were anti-GJA1 (Cx43) antibody (1:250, Abcam #ab11370), anti-GJC1 (Cx45) antibody (1:200, Santa Cruz #sc-374354, Santa Cruz, CA, USA), anti-rabbit anti-YAP antibody (1:100, Cell Signaling #14074), anti-mouse anti-YAP antibody (1:200, Sigma #WH0010413M1), anti-NANOG antibody (1:100, Millipore #AB5731), anti-OCT3/4 antibody (1:100, Santa Cruz, #sc-9081), and anti-SOX2 antibody (1:100, Millipore #AB5603). After washing with 1× PBS three times, cells were incubated with secondary antibodies, either anti-rabbit alexa-488-conjugated antibody (1:200, Jackson ImmunoResearch) or anti-mouse alexa-568-conjugated antibody (1:200, Jackson ImmunoResearch), for 1 h at room temperature, followed by DAPI (Life Technologies, Carlsbad, CA, USA) counterstaining for 10 min at room temperature.

Cells were analyzed using BX-X800 Keyence fluorescence microscope (Keyence, Osaka, Japan) and/or laser confocal microscope (Nikon, Tokyo, Japan).

Signal intensity was measured using ImageJ software by selecting each individual cell and measuring the area, mean intensity, and integrated density. Corrected Total Cell Fluorescence (CTCF) was calculated using the following formula:

CTCF = Integrated Density − (Area of selected cell × Mean fluorescence of background readings).

### 2.6. Apoptosis Assessment Assay

LIVE/DEAD™ Fixable Aqua Dead Cell Stain Kit (Thermo Fisher Scientific, # L34965) was used according to the manufacturer’s instructions.

### 2.7. Cell Cycle Analysis

Samples were collected using trypsin into 1.5 mL microcentrifuge tubes, the cell pellet was washed with 1× PBS, and then the cells were fixed with 70% EtOH for 30 min at 4 °C. After washing, cells were treated with 100 μg/mL PureLink RNase A (Thermo Fisher Scientific, #12091021) for 15 min at 37 °C, followed by PI staining (Thermo Fisher Scientific, #P3566) diluted at 1:1000. Samples were analyzed, using CytoFLEX Flow Cytometer (Beckman Coulter, Brea, CA, USA). Cell cycle analysis was performed using Kaluza Analysis Software (Beckman Coulter).

### 2.8. Dye Transfer Assay

Dye transfer assay was performed as described previously [26].

Briefly, Muse cells were plated onto a 12-well plate at 80% confluency and were incubated overnight. The next day, the culture medium was changed to DDT, TPA, 18α-GA, or DMSO (control) contained medium. Five hundred nM DiIC (Fuji film) was also included in the culture medium described above, and cells were further incubated for 24 h. Muse cells were, afterward, co-cultured with 100 nM Calcein-AM (Dojindo, Kumamoto, Japan)-treated MSC cells for 2.5 h. Finally, co-cultured cells were collected for FACS analysis, using CytoFLEX Flow Cytometer. DiIC-stained cells were detected by phycoerythrin (PE) filter, and Calcein-AM-stained cells were detected by fluorescein isothiocyanate (FITC) filter. Cells with active GJIC appeared as the FITC/PE-double positive population, due to the transfer of Calcein-AM dye into DiIC labeled cells through GJ channels, whereas cells with inactive GJIC were identified as cells positive only for PE.

### 2.9. RNA-Sequencing

Muse cells, plated onto a 12-well plate at 80% confluency, were treated with 90 μM DDT or DMSO control for 24 h and were collected after trypsinization for total RNA collection by using NucleoSpin RNA XS RNA Isolation Kit (Takara Bio), according to the manufacturer’s instructions. Total RNA was, thereafter, subjected to RNA-sequencing by using Illumina HiSeq2500 (San Diego, CA, USA) at a rapid mode. RNA-seq data were mapped using tophat software, and the reads were aligned using hg19 genome database provided by Illumina. Cufflinks was used to detect gene expression levels in the mapped reads, and cuffdiff was used to compare expression levels between samples. Data were then analyzed using multiple data analysis tools, such as DEG analysis, GO analysis, and differential pathway regulation. The following software and tools were used for RNA-seq data analysis: bowtie2, mapping, ver. 2.3.3.1; tophat, mapping, ver. 2.1.1; cufflinks, gene expression, ver. 2.2.1; cuffdiff, relative expression, ver. 2.2.1; samtools, BAM file creation, ver. 0.1.18; UCSC, human genome map, ver. hg19; R-language, ver. 4.0.1; DESeq2, ver. 1.28.1; iDEP, ver. 0.94; Heatmapper, (http://heatmapper.ca/expression/ accessed on 8 April 2022).

cDNA library preparation, sequencing, and reads mapping were performed by the Division of Cell Proliferation at the Tohoku University Graduate School of Medicine.

### 2.10. Cx43 Knockdown

Cx43 siRNA was purchased from Dharmacon Inc. (Lafayette, CO, USA, #M-011042-01-0005), and a 20 μM stock solution was prepared by dissolving in distilled water (Nacalai tesque) following the manufacturer’s instructions and was preserved at −30 °C. A fresh working solution of 2 nM was used in the main experiment.

Muse cells were plated at a density of 20,000 cells/cm^2^, incubated overnight, and then Cx43 siRNA was introduced into Muse cells by using Lipofectamine RNAiMAX (Invitrogen), following the manufacturer’s protocol. Cells were incubated with the transfection medium for 24 h, washed, and then cultured with the culture medium (10% FBS in αMEM with 1 ng/mL bFGF) for 4 days. Total RNA was extracted, and qPCR analysis was performed.

### 2.11. Statistical Analysis

All statistical analyses were performed using Microsoft Excel, Graphpad Prism 9.3.1, or R programming (Version 3.5.1). Student’s *t*-test or Mann-Whitney test was used to assess the significance between two groups, and one-way analysis of variance (ANOVA) followed by Tukey’s multiple comparisons test or Dunnett’s multiple comparisons test was used in the case of three or more groups. The data in this paper were evaluated as mean ± SEM. Statistical significance was determined as follows: ns, not significant *: *p* < 0.05, **: *p* < 0.01, ***: *p* < 0.001, ****: *p* < 0.0001.

## 3. Results

### 3.1. Connexin Gene Expression in Muse and Non-Muse Cells

**Figure 1:**
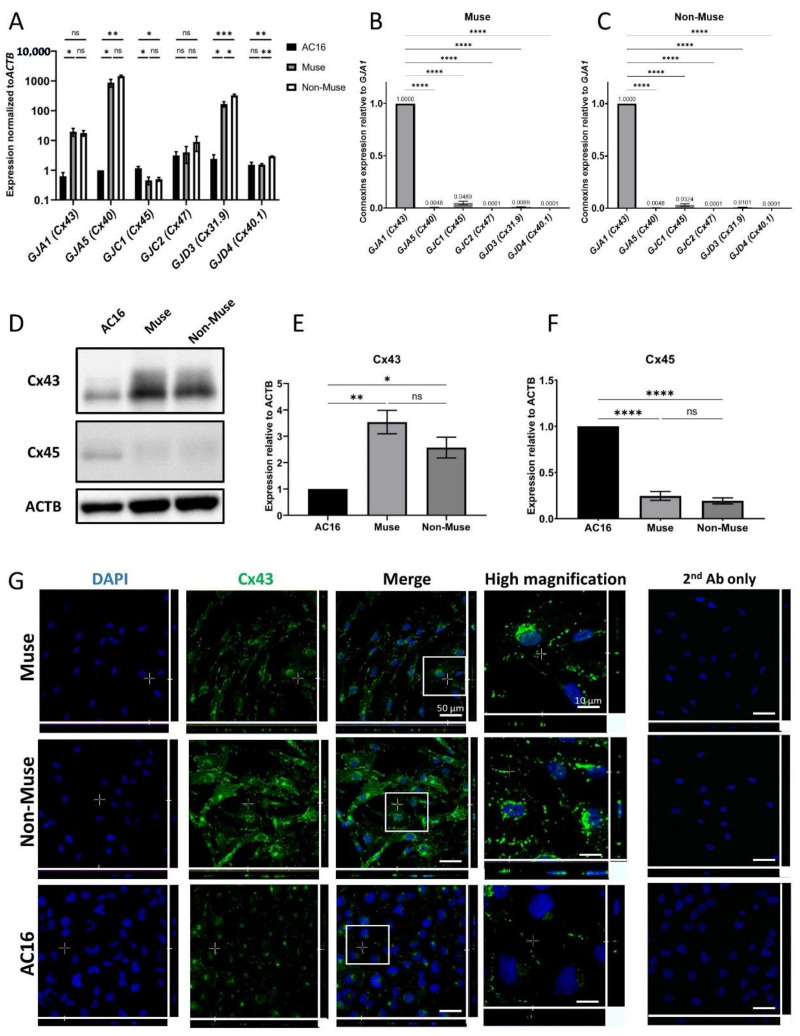
Expression of connexin-encoding genes in human cardiomyocyte, Muse and non-Muse cells, derived from hMSCs. (**A**) qPCR shows the expression of gap junction alpha-1 protein (*GJA1)* and other genes encoding for gap junction proteins in Muse and Non-Muse cells. Human Cardiomyocytes cell line (AC16) cells were used as a positive control (*n* = 3). *: *p* < 0.05, **: *p* < 0.01, ***: *p* < 0.001, ns: no significant difference; (**B**,**C**) The relative expression of connexin encoding gene in Muse cells (**B**), and Non-Muse cells in qPCR (**C**). The expression of each gene was normalized by *ACTB*. The expression level of *GJA1* was set as 1 (*n* = 3). ****: *p* < 0.0001; (**D**–**F**) Western blot of Cx43 and Cx45 in AC16, Muse, and Non-Muse cells. ACTB was used as a housekeeping control. (**E**,**F**) demonstrate the relative expression of Cx43 and Cx45 in Muse and non-Muse cells compared to those in AC16 (*n* = 3). *: *p* < 0.05, **: *p* < 0.01, ****: *p* < 0.0001, ns: no significant difference; (**G**) Laser confocal microscopic images of Cx43 in Muse cells, non-Muse cells, and AC16. DAPI was used for counterstaining. Negative control stained only with 2nd antibody was displayed. Area inside white squares is shown in the high magnification panels. Bars: 50 μm, except for the high magnification fields: 10 μm. One-way ANOVA, followed by Tukey’s multiple comparisons test, was used for statistical analysis in (**A**,**E**,**F**), and one-way ANOVA, followed by Dunnett’s multiple comparisons test, was used in (**B**,**C**). Data are shown as the mean ± SEM.

The SSEA-3-positive Muse cells comprised approximately 5% of the hMSCs, consistent with previous reports [6,15,18]. Muse cells and cells other than Muse cells in hMSCs, namely non-Muse cells that are negative for SSEA-3, showed a limited differentiation capacity as they only differentiated into osteocytes, adipocytes, and chondrocytes, and do not basically express pluripotency genes [15]. They were separated and were subjected to qPCR to analyze the expression of the connexin proteins that form their GJs. Human cardiomyocyte cell line (AC-16) cells were used as a positive control. Muse and Non-Muse cells were shown to express *GJA1*, *GJA5*, *GJC1*, *GJC2*, *GJD3*, and *GJD4* genes that encode Cx43, Cx40, Cx45, Cx47, Cx31.9, and Cx40.1 proteins, respectively. When compared to AC-16, both Muse and non-Muse cells had a higher expression of *GJA5* (*p* < 0.05 for Muse cells and *p* < 0.01 for non-Muse cells), *GJA1* (*p* < 0.05 for Muse cells), and *GJD3* (*p* < 0.05 for Muse cells and *p* < 0.001 for non-Muse cells) (Figure 1A). On the other hand, *GJC1*, *GJC2*, and *GJD4* levels were lower than or at a similar level to those in AC-16 and in Muse and in non-Muse cells (Figure 1A). When compared the expression level of each connexin in Muse cells, *GJA1* was the highest, followed by *GJC1*, *GJD3*, and *GJA5*, with a statistical significance for *GJA1* (*p* < 0.0001) (Figure 1B). Non-Muse cells showed a similar trend (Figure 1C).

We then selected the top two highly expressed connexins, *GJA1* (Cx43) and *GJC1* (Cx45), for Western blot analyses. Cx43 protein level was higher in Muse (*p* < 0.01) and non-Muse cells (*p* < 0.05) when compared to that in AC-16, with statistical significance, however, it did not show statistical significance between Muse cells and non-Muse cells (Figure 1D,E and Supplementary Figure S1). The protein level of Cx45 was, however, higher in AC-16 when compared to that in the Muse and non-Muse cells, (both *p* < 0.0001) with statistical significance (Figure 1D,E and Supplementary Figure S1). The protein levels of Cx45 did not differ between the Muse and non-Muse cells (Figure 1D,F and Supplementary Figure S1).

In immunocytochemistry, Cx43 was located nearby the cell membrane, as dotted signals in the Muse and non-Muse cells (Figure 1G). Cx45 was faintly detected in the Muse and non-Muse cells (Supplementary Figure S2).

In summary, Cx43 was the major connexin expressed in the Muse and non-Muse cells, both in qPCR and Western blot analyses, and thus, we focused on Cx43 in the following experiments.

### 3.2. Chemical Inhibition of GJIC in Muse Cells

GJIC was inhibited either by the treatment of cells with 18α-GA, which reversibly inhibits GJ by altering the connexon particle packing in GJ plaques [27]; TPA that inhibits GJIC via a mitogen-activated protein kinase-extracellular receptor kinase 1/2 (MAPK-ERK1/2)-dependent mechanism; or DDT that inhibits GJ through a phosphatidylcholine-specific phospholipase C (PC-PLC)-dependent mechanism [28,29].

**Figure 2:**
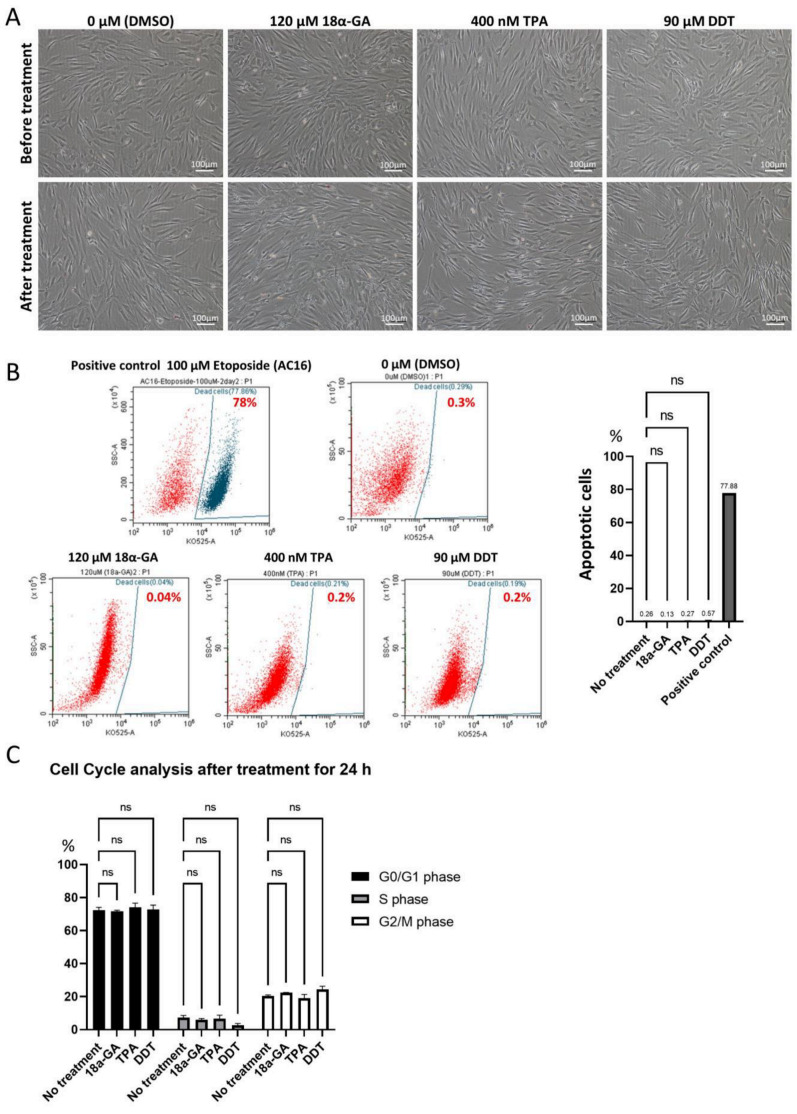
Effect of 18α-GA-, TPA- and DDT-treatments on Muse cells. (**A**) Phase contrast microscopic images of Muse cells before and 24 h after treatment either with 0.1% DMSO (control), 120 μM 18α-GA, 400 nM TPA, or 90 μM DDT. Bars: 100 μm; (**B**) Flow cytometry of Muse cells stained with Fixable Aqua Dead Cell Stain Kit after treatment with DMSO (control), 18α-GA, TPA, and DDT for 24 h. 100 μM Etoposide-treated AC16 cells were used as a positive control. The graph summarizes flow cytometry data, showing the percent of apoptotic cells in each group (*n* = 3). ns: no significant difference. One-way ANOVA, followed by Tukey’s multiple comparisons test, was used for statistical analysis; (**C**) Summary of cell cycle analysis. Bar diagrams show the percent of cells in G0/G1, S, and G2/M phases in untreated Muse cells and Muse cells treated with 18α-GA, TPA, and DDT for 24 h (*n* = 3). ns: no significant difference. One-way ANOVA, followed by Tukey’s multiple comparisons test, was used for statistical analysis; Data are shown as the mean ± SEM.

In order to set usable concentrations for 18α-GA (ranging from 60 to 120 μM), TPA (10 to 400 nM), and DDT (70 to 100 μM), hMSCs were used for the evaluation. Consequently, 24 h incubation with 120 μM 18α-GA, 400 nM TPA, and 90 μM DDT were shown to be less damaging to hMSCs than other concentrations (Supplementary Figure S3). We also confirmed that these concentrations were applicable to Muse cells with no apparent morphological changes (Figure 2A).

The apoptotic cell ratio was ~0.3% in the untreated-Muse cells (cultured under 0.1% DMSO-containing culture medium for 24 h), ~0.1% in 120 μM 18α-GA-, ~0.3% in 400 nM TPA-, and ~0.6% in 90 μM DDT-treated Muse cells after 24 h incubation. All of these data were without statistical significance for the untreated Muse cells but with statistical significance for the positive control (AC-16 cells treated with 100 μM etoposide for 48 h) (Figure 2B). Thus, these three GJ inhibitor treatments did not largely induce apoptosis in the Muse cells.

We next analyzed the effect of the three GJ inhibitors on the cell cycle. Untreated Muse cells in the proportion of 72.3 ± 1.87% were in G0/G1 phase, while 7.3 ± 1.3% were in S phase, and 20.4 ± 0.6% were in G2/M phase. Compared to the untreated Muse cells, the Muse cells treated with 18α-GA, TPA, or DDT did not show significant differences (Figure 2C).

**Figure 3:**
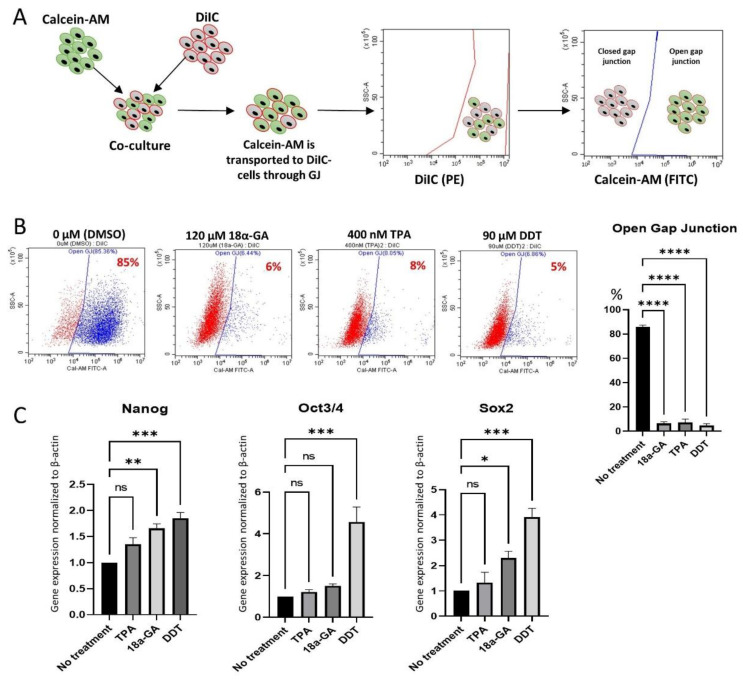
GJ intercellular communication (GJIC) inhibition in Muse cells. (**A**) Schematic diagram of dye transfer assay for confirming GJIC inhibition in Muse cells after treatment either with 18α-GA, TPA, or DDT. Cells were stained with DiIC and Calcein-AM separately, and then they were co-cultured, allowing Calcein-AM to transfer into DiIC-positive cells through GJ. Flow cytometry analysis was performed thereafter, where DiIC/Calcein-AM double-positive cells are representing cells with active GJ, and DiIC-only positive cells are representing GJ inhibited cells; (**B**) Flow cytometry analyses of dye transfer assay in Muse cells 24 h after treatment with each of the three GJ inhibitors. The graph showed the efficient inhibition of GJIC in 18α-GA-, TPA- and DDT-treated Muse cells, with statistical significance (*n* = 3). ****: *p* < 0.0001. One-way ANOVA, followed by Tukey’s multiple comparisons test, was used for statistical analysis. Data are shown as mean ± SEM; (**C**) qPCR analysis of pluripotency markers (*NANOG*, *OCT3/4*, and *SOX2*) after treatment with GJ inhibitors. Each expression level was normalized to *ACTB* (*n* = 3). *: *p* < 0.05, **: *p* < 0.01, ***: *p* < 0.001, ns: no significant difference. One-way ANOVA, followed by Dunnett’s multiple comparisons test, was used for statistical analysis. Data are shown as the mean ± SEM.

Furthermore, the inhibition of GJIC was confirmed by a dye transfer assay, as described previously (Figure 3A) [26]. Approximately 85% of the untreated-Muse cells were double-positive for DilC (indicated by PE) and Calcein-AM (indicated by FITC), suggesting the presence of active GJIC. However, the percentage of DilC/Calcein-AM double-positive cells decreased to ~6%, ~8%, and ~5% in 120 μM 18α-GA-, 400 nM TPA-, and 90 μM DDT-treated Muse cells, respectively (Figure 3A,B). Therefore, GJIC was efficiently inhibited by these inhibitors in the Muse cells.

### 3.3. Effect of GJ Inhibition on Pluripotency Gene Expression

qPCR exhibited up-regulation of the master pluripotency genes, such as *NANOG*, *OCT3/4*, and *SOX2* in Muse cells after treatment with the GJ inhibitors. Compared to the untreated-Muse cells, the elevation of these factors was the most prominent in Muse cells treated with 90 μM DDT, where *NANOG*, *OCT3/4*, and *SOX2* expression levels were 1.85 ± 0.2 (*p* < 0.001), 4.56 ± 1.26 (*p* < 0.001) and 3.92 ± 0.59 (*p* < 0.001) times higher, respectively (Figure 3C).

When Muse cells were treated with 400 nM TPA, *NANOG*, *OCT3/4*, and *SOX2* did not show statistically meaningful changes (Figure 3C). Treatment of the Muse cells with 120 μM 18α-GA exhibited a 1.66 (*p* < 0.01) and a 2.3 (*p* < 0.05) times higher elevation of *NANOG* and *SOX2*, when compared to the untreated Muse cells, respectively, while *OCT3/4* did not show significant changes (Figure 3C).

Since the elevation of the pluripotency gene expression was the most prominent in DDT among the three GJ inhibitors, treatment with 90 μM DDT for 24 h (DDT-Muse cells) was used in the following experiments.

### 3.4. Pluripotency Gene Expression after GJA1 Knockdown in Muse Cells

Cx43 siRNA was used to knock down the *GJA1* gene expression and to observe the gene expression levels of *NANOG*, *OCT3/4*, and *SOX2* in the Muse cells.

**Figure 4:**
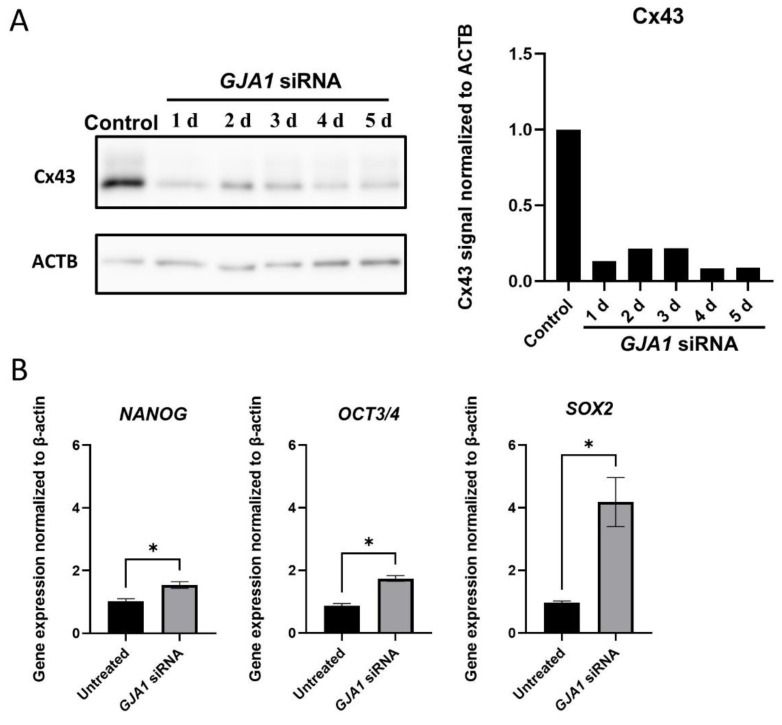
*GJA1* knockdown in Muse cells. (**A**) Western blot of untreated-Muse cells (control), and Muse cells 1–5 days after *GJA1* siRNA transfection. ACTB was used as a housekeeping control. The bar graph shows the protein signal intensity, as quantified using ImageJ software, and normalized to ACTB levels; (**B**) qPCR for pluripotency markers (*NANOG*, *OCT3/4*, and *SOX2*) 4 days after *GJA1* siRNA transfection. Each expression level was normalized by *ACTB* (*n* = 4). *: *p* < 0.05. Mann Whitney test was used for statistical analysis. Data are shown as the mean ± SEM.

siRNA transfection did not induce remarkable morphological changes in the Muse cells (not shown). Effective *GJA1* knockdown was confirmed by a Western blot analysis, which showed that the Cx43 protein levels were down-regulated to 10–20% for up to 5 days after transfection (Figure 4A and Supplementary Figure S4).

A qPCR analysis of pluripotency gene expression showed the significant up-regulation in *NANOG* (1.54 ± 0.21, *p* < 0.05), *OCT3/4* (1.74 ± 0.18, *p* < 0.05), and *SOX2* (4.18 ± 1.57, *p* < 0.05) in the Muse cells 4 days after *GJA1* knockdown when compared to the untreated Muse cells (Figure 4B).

### 3.5. RNA-Sequencing

**Figure 5:**
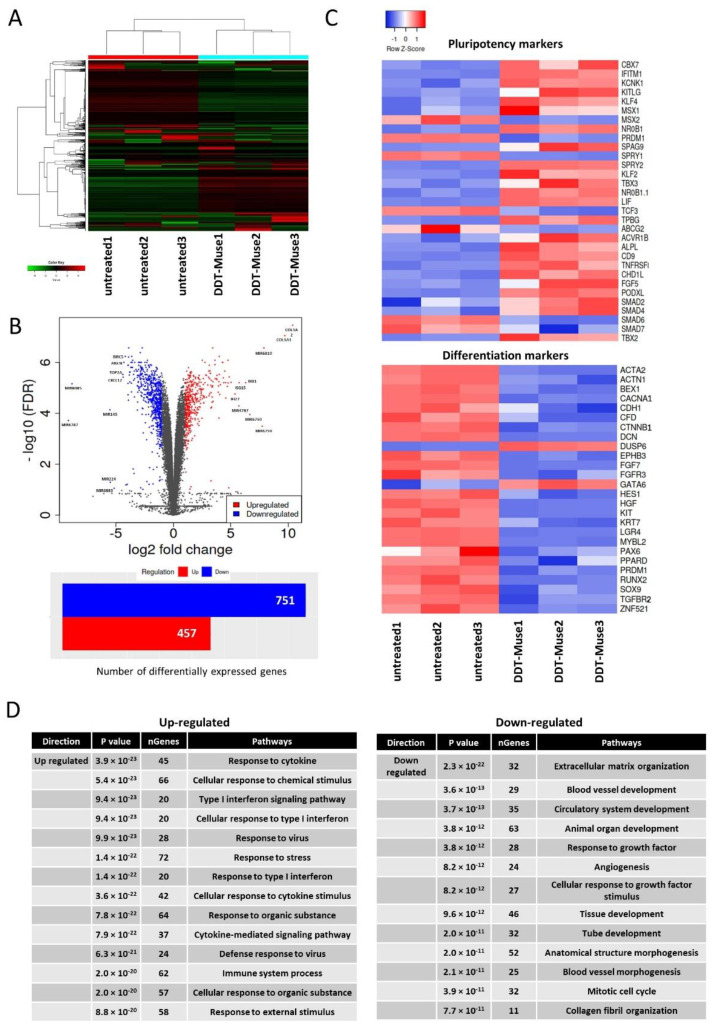
RNA-seq of DDT-treated Muse cells. (**A**) Dendrogram and unsupervised hierarchical clustering heat map of untreated-(1, 2, 3) and DDT-treated (1, 2, 3)-Muse cells. The degree of similarity between gene expression profiles is represented by the vertical distances on the dendrogram. The highest degree of correlation is represented by short vertical distances; (**B**) Volcano plot showing differentially expressed genes between untreated- and DDT-treated-Muse cells. Genes are plotted according to their *p*-value (y-axis), and their expression in DDT-treated cells relative to untreated cells (x-axis) is demonstrated. The number of differentially expressed genes is also shown (Blue: genes down-regulated in DDT-treated Muse cells relative to untreated Muse cells; red: genes up-regulated in DDT-Muse cells relative to untreated Muse cells); (**C**) Heatmaps of genes, relevant to pluripotency and differentiation differentially expressed between untreated-(1, 2, 3) and DDT-treated (1, 2, 3)-Muse cells are shown; (**D**) The list of up-regulated and down-regulated pathways in Muse cells after DDT treatment.

Three replicates of untreated- and DDT-Muse cells were subjected to RNA-sequencing (Figure 5A). A differentially expressed genes (DEG) analysis revealed that 457 genes were up-regulated, and 751 genes were down-regulated in DDT-Muse cells, compared to the untreated-Muse cells (false discovery rate (FDR) < 0.05, fold change > 2) (Figure 5B).

Lineage specification markers, such as alpha-actinin-1 (*ACTN1*), brain-expressed X-linked protein 1 (*BEX1*), P/Q voltage-dependent calcium channel (*CACNA1A*), zinc finger protein 521 (*ZNF521*), hepatocyte growth factor (*HGF*), and tyrosine-protein kinase (*KIT*) were significantly down-regulated, while, pluripotency-related genes, such as kruppel-like factor 4 (*KLF4*), alkaline phosphatase (*ALPL*), chromobox homolog 7 (*CBX7*), sprouty RTK signaling antagonist 2 (*SPRY2*), and *SPRY4* were significantly up-regulated in DDT-Muse cells when compared to the untreated-Muse cells (Figure 5C).

A GO pathway enrichment analysis was performed to identify the top enriched GO biological process terms in DDT treated-Muse cells. Top up-regulated terms included response to cytokine, cellular response to chemical stimulus, type I interferon signaling pathway, and response to organic substance. Down-regulated terms, however, included extracellular matrix organization, blood vessel development, circulatory system development, animal organ development, and response to growth factor (Figure 5D).

### 3.6. Localization of NANOG, OCT3/4, and SOX2 before and after DDT Treatment in Muse Cells

**Figure 6:**
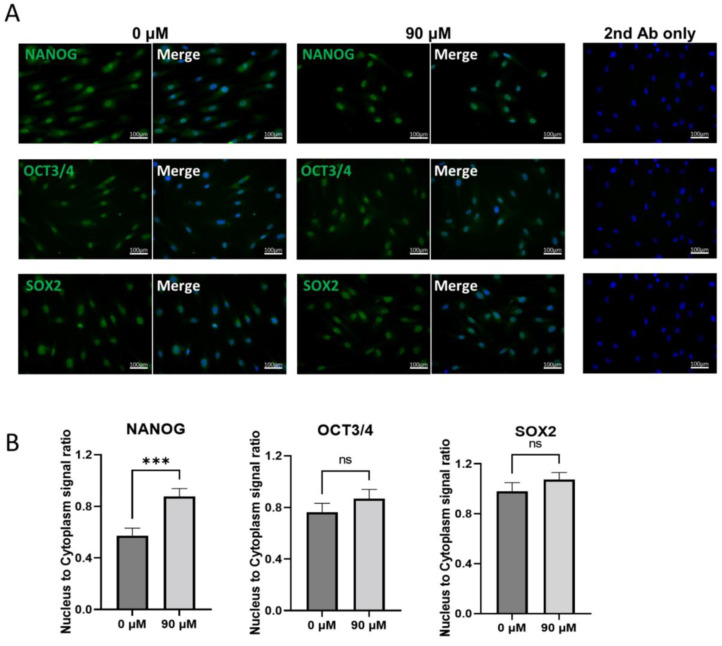
Localization of NANOG, OCT3/4, and SOX2 in Muse cells before and after DDT treatment. (**A**) Immunocytochemistry of untreated Muse cells (0 μM DDT) and DDT-treated Muse cells (90 μM) labeled with NANOG, OCT3/4, or SOX2 (green). Nuclei were labeled with DAPI (blue). Bars: 100 μm; (**B**) The ratio of nucleus to cytoplasm signal intensity of NANOG, OCT3/4, and SOX2 in untreated and DDT-treated Muse cells (*n* = 20 each). Signal intensity was measured using ImageJ software. ***: *p* < 0.001, ns: no significant difference. Mann Whitney test was used for statistical analysis. Data are shown as the mean ± SEM.

The laser confocal microscopic observations of NANOG, OCT3/4, and SOX2 in untreated-Muse cells demonstrated that the signals for those three factors were detected both in the nucleus and cytoplasm, with a higher intensity in the nucleus than in the cytoplasm (Figure 6A). In DDT-Muse cells, however, the absolute signal intensity in the cytoplasm was reduced, and instead, that which was found in the nucleus was elevated, as calculated using a cell fluorescence measurement system, ImageJ software. To evaluate the changes in the signal intensity in the nucleus and cytoplasm, the corrected total intensity ratio of nucleus/cytoplasm was measured and compared between the untreated- and DDT-Muse cells. The nucleus/cytoplasm ratio of NANOG increased 1.66 times in DDT-Muse cells (*p* < 0.001), compared with that in the untreated Muse cells (Figure 6B). The nucleus/cytoplasm ratio of OCT3/4 and SOX2 slightly increased in DDT-Muse cells, compared with that of the untreated-Muse cells, but without statistical significance (Figure 6B).

### 3.7. Localization of Cx43 and YAP in Muse Cells

YAP is a transcription co-activator that acts through binding to TEA domain family member (TEAD) transcription factor [30]. Large tumor suppressor kinase (LATS) and the serine/threonine protein kinase (MST), two core members of the Hippo pathway, phosphorylate YAP and inhibit its transcriptional function by anchoring it to the cytoplasm [30]. It was also reported that YAP can be anchored to the cell membrane through binding to adherens junctions and tight junctions [30]. However, recently, several studies in mouse and rat astrocytes have shown that YAP can also be anchored to the GJ as well [31,32].

**Figure 7:**
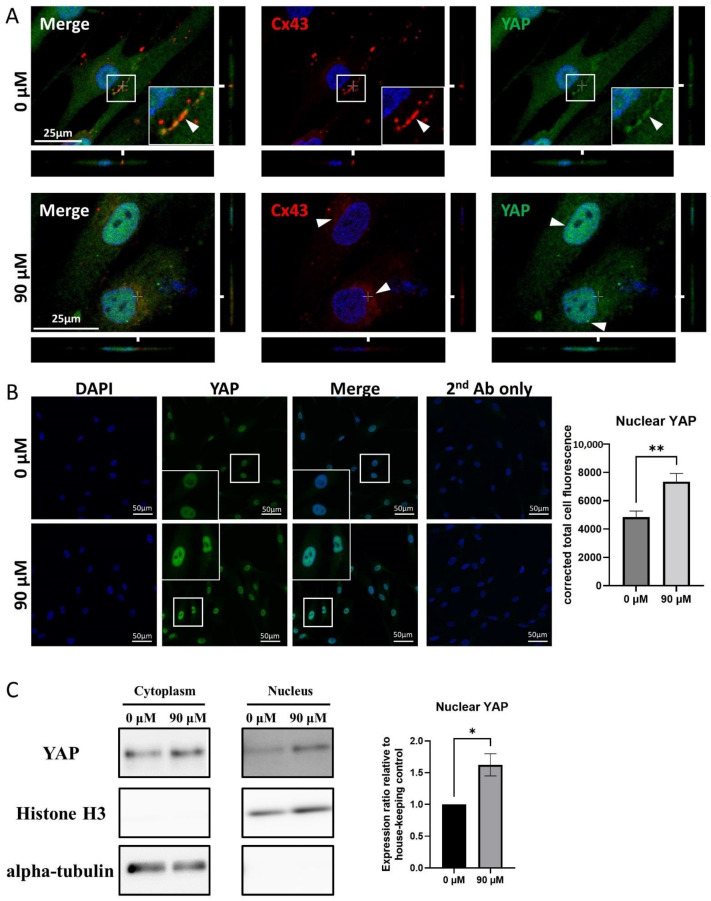
YAP and Cx43 dynamics in Muse cells before and after DDT treatment. (**A**) Immunocytochemistry imaging of Cx43 (red color-coded) and of YAP (green color-coded) in untreated- and DDT-Muse cells. DAPI (blue color-coded) was used for counter-staining. Co-localization of Cx43 and YAP can be observed in the cell membrane of untreated cells (0 μM) (arrowheads). In DDT-treated Muse cells (90 μM), Cx43 signal was faintly detected in the cytoplasm and YAP signal became more intense in the nucleus. Bars: 25 μm; (**B**) Immunocytochemistry imaging of YAP in Muse cells, before and after DDT-treatment. The signal intensity of YAP measured using ImageJ software showed an increase in YAP signal intensity after DDT treatment, with statistical significance (*n* = 30). **: *p* < 0.01. Mann Whitney test was used for statistical analysis. Data are shown as the mean ± SEM; (**C**) Western blot of cytoplasmic and nuclear fractions of untreated and DDT-treated Muse cells. Histone H3 and alpha-tubulin were mainly detected in the nucleus and the cytoplasm, respectively. The intensity of YAP signal in the nucleus was higher in DDT-treated Muse cells, compared to that in untreated Muse cells. Protein signal quantification using ImageJ software showed a significant increase in nuclear YAP signal in DDT-treated Muse cells compared to that in untreated-Muse cells (*n* = 3). *: *p* < 0.05. Student’s *t*-test was used for statistical analysis. Data are shown as the mean ± SEM.

A laser confocal microscopic analysis of Cx43/YAP double staining showed that both signals were adjacent to each other, nearby the cell membrane, in untreated-Muse cells (Figure 7A). However, after DDT treatment, the Cx43 signal was faintly detected in the cytoplasm and the YAP signal became more intense in the nucleus, compared to that in the untreated cells (Figure 7A).

A corrected signal intensity measurement demonstrated that the nuclear YAP intensity was higher in DDT-Muse cells rather than in untreated-Muse cells, with statistical significance (*p* < 0.01) (Figure 7B).

A Western blot analysis exhibited the elevated YAP signal in the nuclei of DDT-Muse cells, compared to that in untreated-Muse cells, as shown by the quantification of protein bands using ImageJ software (*p* < 0.05). Histone H3, the nuclear loading control, was detected mainly in the nucleus, and α-tubulin, the cytoplasmic loading control, was detected in the cytoplasm (Figure 7C, Supplementary Figures S5 and S6).

## 4. Discussion

**Figure 8:**
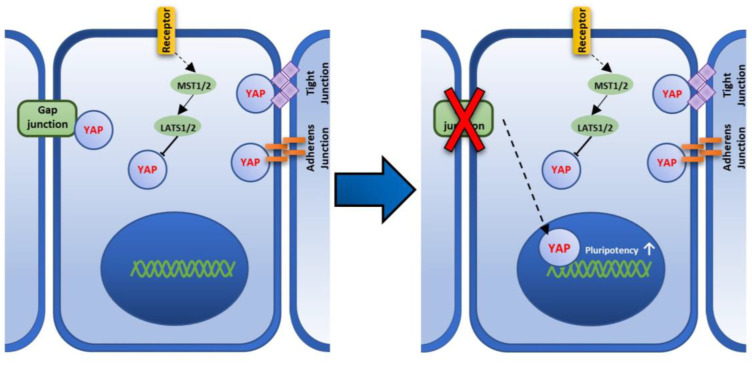
A figure showing the proposed Cx43-YAP mediated pathway that regulates pluripotency in Muse cells. Hippo pathway that is mediated by large tumor suppressor kinase (LATS) and the serine/threonine protein kinase (MST), phosphorylates Yes-associated protein (YAP), and inhibits its transcriptional function by anchoring it to the cytoplasm [30]. YAP is also anchored to the cell membrane through binding to the gap junction, adherens junction, and tight junction [30]. Upon GJ disruption, YAP is translocated to the nucleus leading to the up-regulation of pluripotency factors.

Muse cells are endogenous pluripotent stem cells and are non-tumorigenic, but their expression levels of pluripotency master genes, such as Nanog, Oct3/4, and Sox2 in the adherent culture system, are lower than those in the suspension culture. When Muse cells are transferred to a suspension culture, the size of each cell becomes smaller and they form a sphere-shaped cluster. In this condition, the expression levels of Nanog, Oct3/4, and Sox2 are increased and become higher than those in an adherent culture [33]. ES cells and iPSCs were also shown to up-regulate the pluripotency genes in a suspension culture [7,13,24,25]. Thus, suspension culture is one of the simple approaches used to up-regulate the pluripotency gene expression in these stem cells. On the other hand, methods to up-regulate pluripotency genes in adherent culture system have not been reported in Muse cells, except for the induction of iPSCs by introducing Yamanaka-four factors [15]. In this study, we propose that Cx43, a component of GJ, and YAP, a mechanosensory factor, play an important role in pluripotency regulation and that the disruption of Cx43-mediated GJIC up-regulates the master pluripotency genes, *NANOG*, *OCT3/4*, and *SOX2*, in an adherent culture system in Muse cells. The possible mechanism is described in Figure 8.

GJ channels mediate intercellular communication in multicellular organisms and play an important role in regulating cell growth and differentiation [34,35]. This important role can also be observed in cancer cells as the process of carcinogenesis is usually accompanied by the down-regulation of connexin expression, suggesting that the lack of communication between cells disrupts their homogeneity, allowing the appearance of abnormal cells with higher pluripotency and proliferation, namely cancer stem cells [34,35,36,37].

In this study, we found that *GJA1* was the highest connexin subtype expressed in pluripotent-like Muse cells. *GJA5*, *GJC1*, *GJC2*, I, and *GJD4* expression was also detected byqPCR, although to a far lesser extent than *GJA1*. Western blot and immunocytochemistry analyses supported these data. Therefore, we focused on *GJA1* that encodes the Cx43 protein and analyzed its role in pluripotency regulation in Muse cells. Three GJ inhibitors, 18α-GA, TPA, and DDT, were chosen to block Cx43-mediated GJIC in Muse cells and the efficiency of GJIC inhibition after treatment with each inhibitor was confirmed by dye-transfer assay.

qPCR analysis of 18α-GA-, TPA- and DDT-treated-Muse cells showed that the expression levels of *NANOG*, *OCT3/4*, and *SOX2* were increased—or showed a tendency of up-regulation—in all three kinds of inhibitors, when compared to the untreated-Muse cells. Although all of the three inhibitors effectively inhibited GJIC in Muse cells, their influence on pluripotency up-regulation was different from each other; DDT had the highest and most significant up-regulation of *NANOG*, *OCT3/4*, and *SOX2*; 18α-GA had the significant up-regulation of *NANOG*, and *SOX2*; TPA had a tendency of up-regulation but with no statistical significance in *NANOG*, *OCT3/4*, and *SOX2*. One of the reasons for these different responses might be due to the various mechanisms of GJ inhibition these inhibitors have. DDT causes actual GJ disruption by internalizing Cx43 into the cytoplasm and its degradation [28]. TPA, on the other hand, mainly inhibits GJIC through Cx43 hyperphosphorylation [38], while 18a-GA affects the membrane fluidity, disrupting the individual GJ units to assemble and form a GJ plaque [27]. The action of DDT for GJ inhibition might be more direct and efficient than TPA and 18a-GA.

*GJA1* knockdown by siRNA transfection also showed the up-regulation of pluripotency genes in Muse cells, supporting the results of GJ inhibitor experiments.

RNA-seq data showed a difference in gene expression pattern between naïve- and DDT-treated Muse cells, with 457 up-regulated and 751 down-regulated genes in DDT-treated Muse cells. A pathway analysis showed an overall down-regulation of the cell differentiation-related pathways (e.g., blood vessel development, organ and tissue development). These data are consistent with previous reports suggesting that Cx43 is positively correlated with differentiation and negatively correlated with pluripotency gene expression in stem cells since GJIC plays a role in the differentiation of pluripotent stem cells and Cx43 levels are elevated during differentiation into many different cell types [39,40,41,42].

Hippo pathway plays an evolutionarily conserved role in organ size control by inhibiting cell proliferation, promoting apoptosis, and regulating the fates of stem/progenitor cells [43]. YAP, which is related to Hippo pathway activity, is a protein that acts as a co-transcriptional regulator by activating the transcription process of genes involved in cell proliferation and apoptotic gene suppression [44]. YAP is inhibited through Hippo pathway signaling and only exhibits its transcriptional effects after translocating into the nucleus upon the inhibition of Hippo pathway signaling [45].

YAP has another important aspect. It is a primary sensor of the cell’s physical nature, e.g., cell structure, shape, and polarity [43]. YAP activation reflects a cell’s “social” behavior through cell adhesion and the mechanical signals that are perceived from the tissue architecture and the surrounding extracellular matrix [43]. Recent studies reported that YAP is physically associated with Cx43, and upon Cx43 disruption, YAP would translocate to the nucleus of mouse and rat astrocytes [31,32]. Similar to those observations, untreated Muse cells exhibited that YAP, co-localized with Cx43 in the cell membrane, dissociated from the cell membrane after DDT-treatment, as shown by immunocytochemistry imaging, and translocated to the nucleus, because the nucleic YAP signal intensity was significantly higher in the immunocytochemistry and Western blot analyses after DDT-treatment. Thus, it is possible that, when GJ connection is disrupted, YAP is translocated into the nucleus, leading to the activation of its co-transcriptional activity. Concomitantly, as mentioned above, an overall, down-regulation of cell differentiation-related pathways was observed.

It is reported that YAP and TEAD2 were highly expressed in self-renewing mouse ES cells and that YAP expression is up-regulated during iPSC reprogramming [44,45]. Indeed, TEAD2 associates directly with the promoter of OCT3/4, one of the master pluripotency genes [46]. Reversely, YAP gene silencing was reported to induce the loss of pluripotency in ES cells [47]. These reports indicate the important role of YAP in pluripotency regulation [46,48].

In this study, we focused on the role of Cx43 in Muse cells treated with these three different GJIC inhibitors that work by very different biochemical mechanisms to inhibit functional GJIC at the posttranscriptional level, as well as in *GJA1* siRNA-transfected Muse cells. Other studies, using iPS cells (Cx43 knockout (*GJA1*^−/−^)) iPSCs, generated using CRISPR-Cas9 gene ablation), maintained characteristics typical of iPSCs and successfully differentiated into cells of ectoderm, mesoderm, or endoderm lineages [49]. While these studies were well executed, the interpretation of these results is complicated and might not apply to normal adult organ-specific adult stem cells or normal Muse cells. These iPS cells’ origin has been questioned, in that they might be derived from normal fibroblast stem cells rather than having been “reprogramed” [50,51].

These iPS cells have both the endogenous *OCT4* gene, as well as the extra copy of the exogenous *OCT4* gene. By definition, these iPS cells, when injected into an adult animal, form teratomas or the three primary germ lines. Whereas the organ-specific adult stem cells do not form teratomas. The normal human adult stem cells, i.e., human breast stem cells, do not express *GJA1*, nor do they have functional GJIC [52]. When these normal human breast stem cells are induced to differentiate into human breast epithelial cells, *OCT4* is transcriptionally repressed, *GJA1* is expressed, and functional GJIC is evident. If, however, the normal adult breast stem cells are transfected with the Large T antigen gene of SV40 virus, the cells do not express *GJA1*, maintain expression of *OCT4*, and do not differentiate. They stay in the stem cell state or remain “immortal”. Clearly, further studies must be done to clarify the exact mechanism of how YAP interacts with the promoters of pluripotency related-genes in these cells, as well as the differences between these Muse cells and iPS cells in the future.

The expression of *GJA1* is different between naïve and primed pluripotent states. Human primed pluripotent stem cells express higher *GJA1* than they do in the naïve state [23], and naïve pluripotent stem cells are less affected by the pharmacological ablation of GJIC than primed pluripotent stem cells are [23]. Muse cells were suggested to be more similar to primed pluripotent stem cells than to naïve pluripotent stem cells for their proliferation and self-renewability and are ultimately dependent on the basic fibroblast growth factor (bFGF), rather than the leukemia inhibitory factor (LIF) [5]. The result of this study, namely the expression of *GJA1* and the responsiveness of pluripotency gene expression by GJ inhibition, supports the assumption that Muse cells are similar to primed pluripotent stem cells.

While we demonstrated the involvement of a Cx43/YAP system in the up-regulation of pluripotency genes, *NANOG*, *OCT3/4*, and *SOX2*, when GJIC was inhibited, other systems might also be involved in this phenomenon. Furthermore, multiple steps/systems might intervene in the inhibition of GJ and pluripotency gene up-regulation. These interesting subjects need to be clarified in future studies.

## Data Availability

Not applicable.

## Supplementary Materials

The following supporting information can be downloaded at: https://www.mdpi.com/article/10.3390/cells11172701/s1, Figure S1: Western blot analysis of Cx43 and Cx45 in human cardiomyocyte, Muse, and non-Muse cells derived from hMSC.; Figure S2. Cx45 immunostaining in human cardiomyocyte, Muse, and non-Muse cells derived from hMSC; Figure S3. hMSC morphology after treatment with 18α-GA, TPA, and DDT for 24 h; Figure S4. Western blot analysis of Cx43 in GJA1-knockdown Muse cells; Figure S5. Western blot analysis of nuclear YAP in untreated- and DDT-Muse cells; Figure S6. Western blot analysis of cytoplasmic YAP in untreated- and DDT-Muse cells.

## References

[1] Laird D.W. (2006). Life cycle of connexins in health and disease. Biochem. J..

[2] Goodenough D.A., Paul D.L. (2009). Gap Junctions. Cold Spring Harb. Perspect. Biol..

[3] Worsdorfer P., Bosen F., Gebhardt M., Russ N., Zimmermann K., Kessie D.K., Sekaran T., Egert A., Ergun S., Schorle H. (2017). Abrogation of Gap Junctional Communication in ES Cells Results in a Disruption of Primitive Endoderm Formation in Embryoid Bodies. Stem Cells.

[4] Sharovskaya Y.Y., Philonenko E.S., Kiselev S.L., Lagarkova M.A. (2012). De Novo Reestablishment of Gap Junctional Intercellular Communications During Reprogramming to Pluripotency and Differentiation. Stem Cells Dev..

[5] Wakao S., Kushida Y., Dezawa M., Dezawa M. (2018). Basic Characteristics of Muse Cells. Muse Cells: Endogenous Reparative Pluripotent Stem Cells.

[6] Kuroda Y., Kitada M., Wakao S., Nishikawa K., Tanimura Y., Makinoshima H., Goda M., Akashi H., Inutsuka A., Niwa A. (2010). Unique multipotent cells in adult human mesenchymal cell populations. Proc. Natl. Acad. Sci. USA.

[7] Sato T., Wakao S., Kushida Y., Tatsumi K., Kitada M., Abe T., Niizuma K., Tominaga T., Kushimoto S., Dezawa M. (2020). A Novel Type of Stem Cells Double-Positive for SSEA-3 and CD45 in Human Peripheral Blood. Cell Transplant..

[8] Alessio N., Ozcan S., Tatsumi K., Murat A., Peluso G., Dezawa M., Galderisi U. (2017). The secretome of MUSE cells contains factors that may play a role in regulation of stemness, apoptosis and immunomodulation. Cell Cycle.

[9] Acar M.B., Aprile D., Ayaz-Guner S., Guner H., Tez C., Di Bernardo G., Peluso G., Ozcan S., Galderisi U. (2021). Why Do Muse Stem Cells Present an Enduring Stress Capacity? Hints from a Comparative Proteome Analysis. Int. J. Mol. Sci..

[10] Aprile D., Alessio N., Demirsoy I.H., Squillaro T., Peluso G., Di Bernardo G., Galderisi U. (2021). MUSE Stem Cells Can Be Isolated from Stromal Compartment of Mouse Bone Marrow, Adipose Tissue, and Ear Connective Tissue: A Comparative Study of Their In Vitro Properties. Cells.

[11] Ogura F., Wakao S., Kuroda Y., Tsuchiyama K., Bagheri M., Heneidi S., Chazenbalk G., Aiba S., Dezawa M. (2014). Human adipose tissue possesses a unique population of pluripotent stem cells with nontumorigenic and low telomerase activities: Potential implications in regenerative medicine. Stem Cells Dev..

[12] Uchida H., Niizuma K., Kushida Y., Wakao S., Tominaga T., Borlongan C.V., Dezawa M. (2017). Human Muse Cells Reconstruct Neuronal Circuitry in Subacute Lacunar Stroke Model. Stroke.

[13] Amin M., Kushida Y., Wakao S., Kitada M., Tatsumi K., Dezawa M. (2018). Cardiotrophic Growth Factor-Driven Induction of Human Muse Cells into Cardiomyocyte-Like Phenotype. Cell Transplant..

[14] Tsuchiyama K., Wakao S., Kuroda Y., Ogura F., Nojima M., Sawaya N., Yamasaki K., Aiba S., Dezawa M. (2013). Functional melanocytes are readily reprogrammable from multilineage-differentiating stress-enduring (muse) cells, distinct stem cells in human fibroblasts. J. Investig. Dermatol..

[15] Wakao S., Kitada M., Kuroda Y., Shigemoto T., Matsuse D., Akashi H., Tanimura Y., Tsuchiyama K., Kikuchi T., Goda M. (2011). Multilineage-differentiating stress-enduring (Muse) cells are a primary source of induced pluripotent stem cells in human fibroblasts. Proc. Natl. Acad. Sci. USA.

[16] Yamauchi T., Yamasaki K., Tsuchiyama K., Koike S., Aiba S. (2017). The Potential of Muse Cells for Regenerative Medicine of Skin: Procedures to Reconstitute Skin with Muse Cell-Derived Keratinocytes, Fibroblasts, and Melanocytes. J. Investig. Dermatol..

[17] Uchida H., Morita T., Niizuma K., Kushida Y., Kuroda Y., Wakao S., Sakata H., Matsuzaka Y., Mushiake H., Tominaga T. (2016). Transplantation of Unique Subpopulation of Fibroblasts, Muse Cells, Ameliorates Experimental Stroke Possibly via Robust Neuronal Differentiation. Stem Cells.

[18] Katagiri H., Kushida Y., Nojima M., Kuroda Y., Wakao S., Ishida K., Endo F., Kume K., Takahara T., Nitta H. (2016). A Distinct Subpopulation of Bone Marrow Mesenchymal Stem Cells, Muse Cells, Directly Commit to the Replacement of Liver Components. Am. J. Transplant..

[19] Uchida N., Kushida Y., Kitada M., Wakao S., Kumagai N., Kuroda Y., Kondo Y., Hirohara Y., Kure S., Chazenbalk G. (2017). Beneficial Effects of Systemically Administered Human Muse Cells in Adriamycin Nephropathy. J. Am. Soc. Nephrol..

[20] Yamada Y., Wakao S., Kushida Y., Minatoguchi S., Mikami A., Higashi K., Baba S., Shigemoto T., Kuroda Y., Kanamori H. (2018). S1P-S1PR2 Axis Mediates Homing of Muse Cells into Damaged Heart for Long-Lasting Tissue Repair and Functional Recovery After Acute Myocardial Infarction. Circ. Res..

[21] Noda T., Nishigaki K., Minatoguchi S. (2020). Safety and Efficacy of Human Muse Cell-Based Product for Acute Myocardial Infarction in a First-in-Human Trial. Circ. J..

[22] Fujita Y., Nohara T., Takashima S., Natsuga K., Adachi M., Yoshida K., Shinkuma S., Takeichi T., Nakamura H., Wada O. (2021). Intravenous allogeneic multilineage-differentiating stress-enduring cells in adults with dystrophic epidermolysis bullosa: A phase 1/2 open-label study. J. Eur. Acad. Dermatol. Venereol..

[23] Esseltine J.L., Brooks C.R., Edwards N.A., Subasri M., Sampson J., Seguin C., Betts D.H., Laird D.W. (2020). Dynamic regulation of connexins in stem cell pluripotency. Stem Cells.

[24] Nath S.C., Day B., Harper L., Yee J., Hsu C.Y.M., Larijani L., Rohani L., Duan N., Kallos M.S., Rancourt D.E. (2021). Fluid shear stress promotes embryonic stem cell pluripotency via interplay between beta-catenin and vinculin in bioreactor culture. Stem Cells.

[25] Yoda K., Ohnuki Y., Masui S., Kurosawa H. (2020). Optimized conditions for the supplementation of human-induced pluripotent stem cell cultures with a GSK-3 inhibitor during embryoid body formation with the aim of inducing differentiation into mesodermal and cardiac lineage. J. Biosci. Bioeng..

[26] Fonseca P.C., Nihei O.K., Savino W., Spray D.C., Alves L.A. (2006). Flow cytometry analysis of gap junction-mediated cell-cell communication: Advantages and pitfalls. Cytom. Part A.

[27] Goldberg G.S., Moreno A.P., Bechberger J.F., Hearn S.S., Shivers R.R., MacPhee D.J., Zhang Y.C., Naus C.C.G. (1996). Evidence that disruption of connexon particle arrangements in gap junction plaques is associated with inhibition of gap junctional communication by a glycyrrhetinic acid derivative. Exp. Cell Res..

[28] Sovadinova I., Babica P., Boke H., Kumar E., Wilke A., Park J.S., Trosko J.E., Upham B.L. (2015). Phosphatidylcholine Specific PLC-Induced Dysregulation of Gap Junctions, a Robust Cellular Response to Environmental Toxicants, and Prevention by Resveratrol in a Rat Liver Cell Model. PLoS ONE.

[29] Babica P., Ctverackova L., Lencesova Z., Trosko J.E., Upham B.L. (2016). Chemopreventive Agents Attenuate Rapid Inhibition of Gap Junctional Intercellular Communication Induced by Environmental Toxicants. Nutr. Cancer Int. J..

[30] Ramos A., Camargo F.D. (2012). The Hippo signaling pathway and stem cell biology. Trends Cell Biol..

[31] Yang Y., Ren J., Sun Y.H., Xue Y., Zhang Z.J., Gong A.H., Wang B.F., Zhong Z.H., Cui Z.W., Xi Z.Y. (2018). A connexin43/YAP axis regulates astroglial-mesenchymal transition in hemoglobin induced astrocyte activation. Cell Death Differ..

[32] Yu H.L., Cao X., Li W., Liu P.Y., Zhao Y.Y., Song L.L., Chen J., Chen B.L., Yu W.K., Xu Y. (2020). Targeting connexin 43 provides anti-inflammatory effects after intracerebral hemorrhage injury by regulating YAP signaling. J. Neuroinflamm..

[33] Iseki M., Kushida Y., Wakao S., Akimoto T., Mizuma M., Motoi F., Asada R., Shimizu S., Unno M., Chazenbalk G. (2017). Human Muse Cells, Nontumorigenic Pluripotent-Like Stem Cells, Have Liver Regeneration Capacity Through Specific Homing and Cell Replacement in a Mouse Model of Liver Fibrosis. Cell Transplant..

[34] Loewenstein W. (1979). Junctional intercellular communication and the control of growth. Biochim. Biophys. Acta BBA Rev. Cancer.

[35] Yamasaki H., Naus C.C.G. (1996). Role of connexin genes in growth control. Carcinogenesis.

[36] Mesnil M., Crespin S., Avanzo J.L., Zaidan-Dagli M.L. (2005). Defective gap junctional intercellular communication in the carcinogenic process. Biochim. Biophys. Acta Biomembr..

[37] Trosko J.E. (2003). The role of stem cells and gap junctional intercellular communication in carcinogenesis. J. Biochem. Mol. Biol..

[38] Rivedal E., Opsahl H. (2001). Role of PKC and MAP kinase in EGF- and TPA-induced connexin43 phosphorylation and inhibition of gap junction intercellular communication in rat liver epithelial cells. Carcinogenesis.

[39] Talukdar S., Emdad L., Das S.K., Fisher P.B. (2022). GAP junctions: Multifaceted regulators of neuronal differentiation. Tissue Barriers.

[40] Koizumi J.I., Kojima T., Kamekura R., Kurose M., Harimaya A., Murata M., Osanai M., Chiba H., Himi T., Sawada N. (2007). Changes of gap and tight junctions during differentiation of human nasal epithelial cells using primary human nasal epithelial cells and primary human nasal fibroblast cells in a noncontact coculture system. J. Membr. Biol..

[41] Gu S.M., Yu X.S., Yin X.Y., Jiang J.X. (2003). Stimulation of lens cell differentiation by gap junction protein connexin 45.6. Investig. Ophthalmol. Vis. Sci..

[42] Hirschi K.K., Burt J.M., Hirschi K.D., Dai C.P. (2003). Gap junction communication mediates transforming growth factor-beta activation and endothelial-induced mural cell differentiation. Circ. Res..

[43] Piccolo S., Dupont S., Cordenonsi M. (2014). The Biology of Yap/Taz: Hippo Signaling and Beyond. Physiol. Rev..

[44] Chen Y.A., Lu C.Y., Cheng T.Y., Pan S.H., Chen H.F., Chang N.S. (2019). WW Domain-Containing Proteins YAP and TAZ in the Hippo Pathway as Key Regulators in Stemness Maintenance, Tissue Homeostasis, and Tumorigenesis. Front. Oncol..

[45] Xu Y.X., Wang X.Y., Yu M., Ruan Y., Zhang J.L., Tian Y.P., Xiong J.X., Liu L.L., Cheng Y.D., Yang Y. (2021). Identification, subcellular localization, and functional comparison of novel Yap splicing isoforms in mouse embryonic stem cells. Iubmb Life.

[46] Tamm C., Bower N., Anneren C. (2011). Regulation of mouse embryonic stem cell self-renewal by a Yes-YAP-TEAD2 signaling pathway downstream of LIF. J. Cell Sci..

[47] Wang X.Y., Ruan Y., Zhang J.L., Tian Y.P., Liu L.L., Wang J.L., Liu G.K., Cheng Y.D., Xu Y.X., Yang Y. (2021). Expression levels and activation status of Yap splicing isoforms determine self-renewal and differentiation potential of embryonic stem cells. Stem Cells.

[48] Lian I., Kim J., Okazawa H., Zhao J.G., Zhao B., Yu J.D., Chinnaiyan A., Israel M.A., Goldstein L.S.B., Abujarour R. (2010). The role of YAP transcription coactivator in regulating stem cell self-renewal and differentiation. Genes Dev..

[49] Christopher G.A., Noort R.J., Esseltine J.L. (2022). Connexin 43 Gene Ablation Does Not Alter Human Pluripotent Stem Cell Germ Lineage Specification. Biomolecules.

[50] Trosko J.E. (2008). Commentary: “Re-programming or selecting adult stem cells?”. Stem Cell Rev..

[51] Trosko J.E. (2021). On the potential origin and characteristics of cancer stem cells. Carcinogenesis.

[52] Tai M.H., Chang C.C., Olson L.K., Trosko J.E. (2005). Oct4 expression in adult human stem cells: Evidence in support of the stem cell theory of carcinogenesis. Carcinogenesis.

