# Supplementary material for: Inhibition of Gap Junctional Intercellular Communication Upregulates Pluripotency Gene Expression in Endogenous Pluripotent Muse Cells
Source: Cells. 2022 Aug 30;11(17):2701. doi: 10.3390/cells11172701 (PMC9455024; doi:10.3390/cells11172701)
Supplement: Supplementary file 1 [file cells-11-02701-s001.zip › cells-1701890-supplementary.pdf]

Supplementary figures

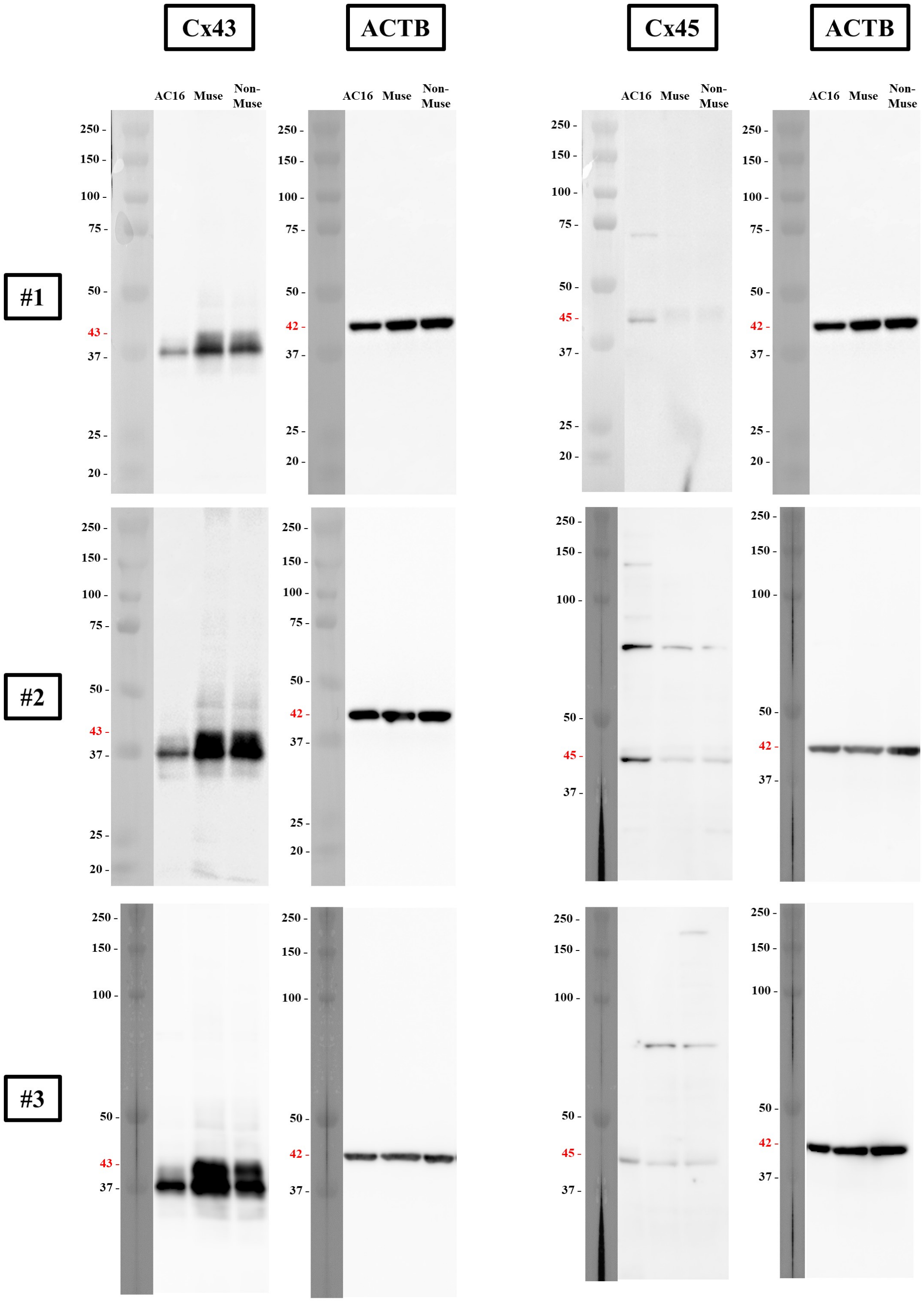

Figure S1. Western blot analysis of Cx43 and Cx45 in human cardiomyocytes, Muse, and non-Muse cells derived from hMSC.

Images showing full membranes used for western blot analysis represented in figures 1D, 1E, and 1F.

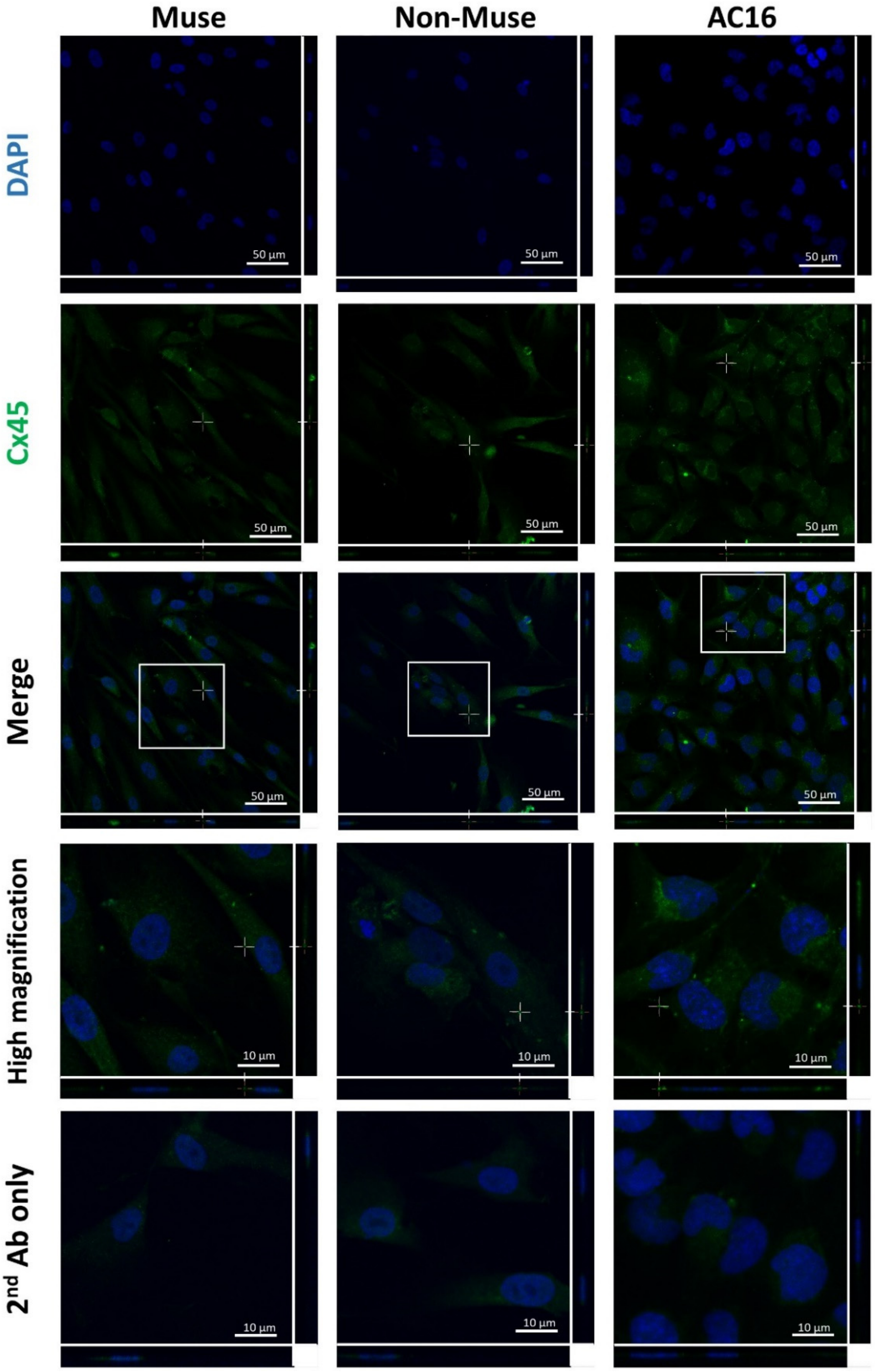

Figure S2. Cx45 immunostaining in human cardiomyocyte, Muse, and non-Muse cells derived from hMSC.

Laser confocal microscopic images of Cx43 in Muse cells, non-Muse cells, and AC16. DAPI was used for counter-staining. Negative control stained only with 2nd antibody is displayed. Area inside white squares is shown in the high magnification panels. Bars: 50 μm, except for the high magnification fields and 2nd Ab only fields: 10 μm.

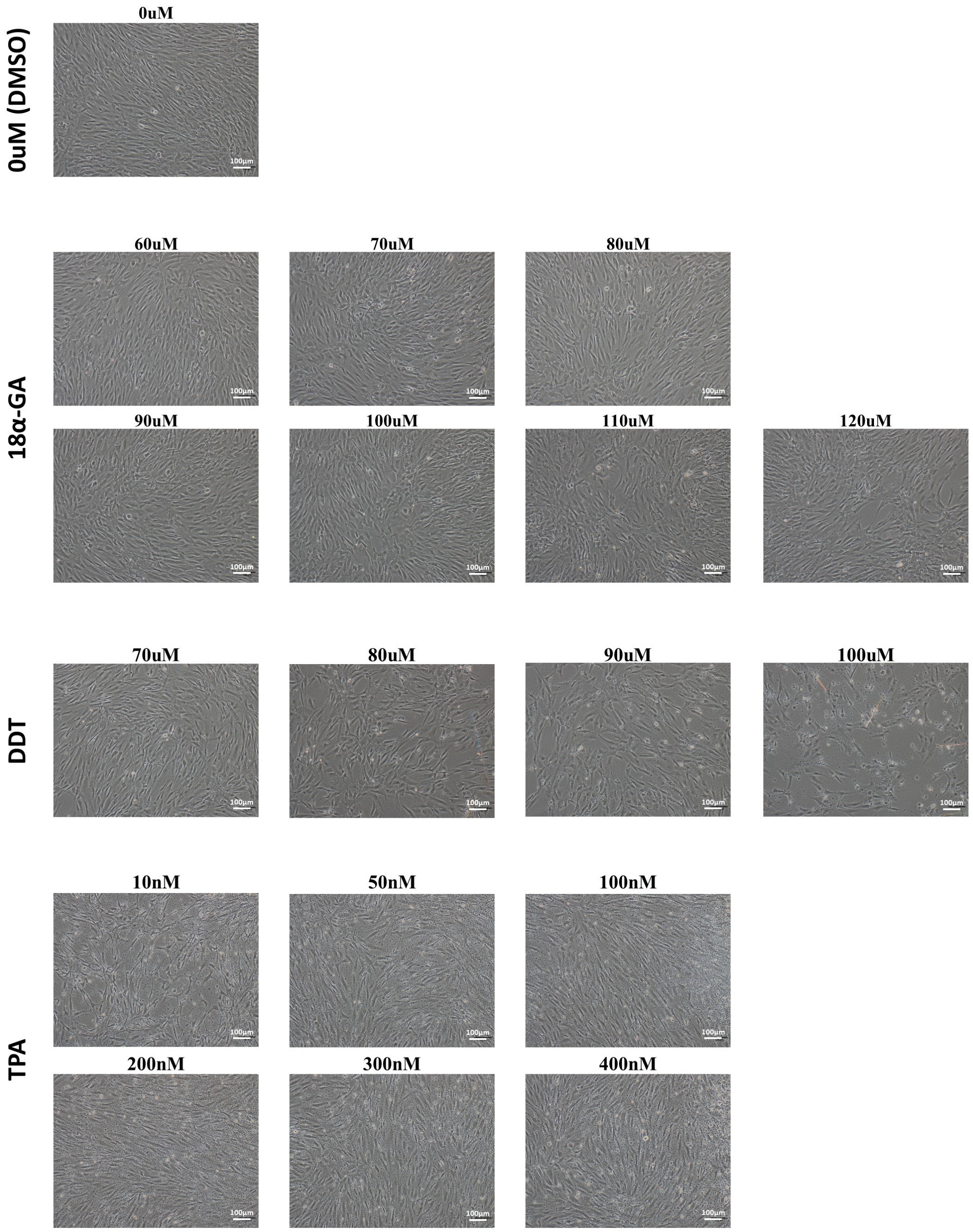

Figure S3. hMSC morphology after treatment with 18α-GA, TPA, and DDT for 24 h.

Phase contrast microscopic images of hMSC cells before and 24 hours after treatment either with 0.1% DMSO (control), 18α-GA (60 to 120 μM), TPA (10 to 400 nM), and DDT (70 to 100 μM). Bars: 100 **μm.**

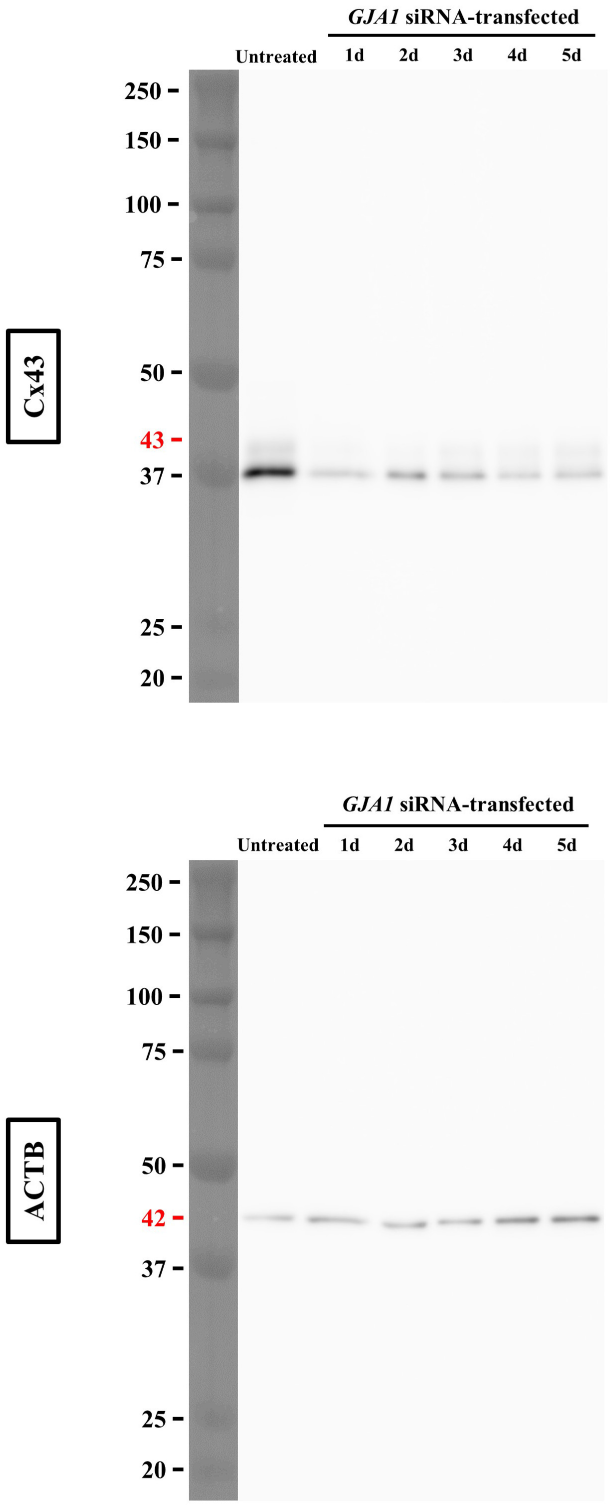

Figure S4. Western blot analysis of Cx43 in *GJA1*-knockdown Muse cells.

Images showing full membranes used for western blot analysis represented in figure 4A.

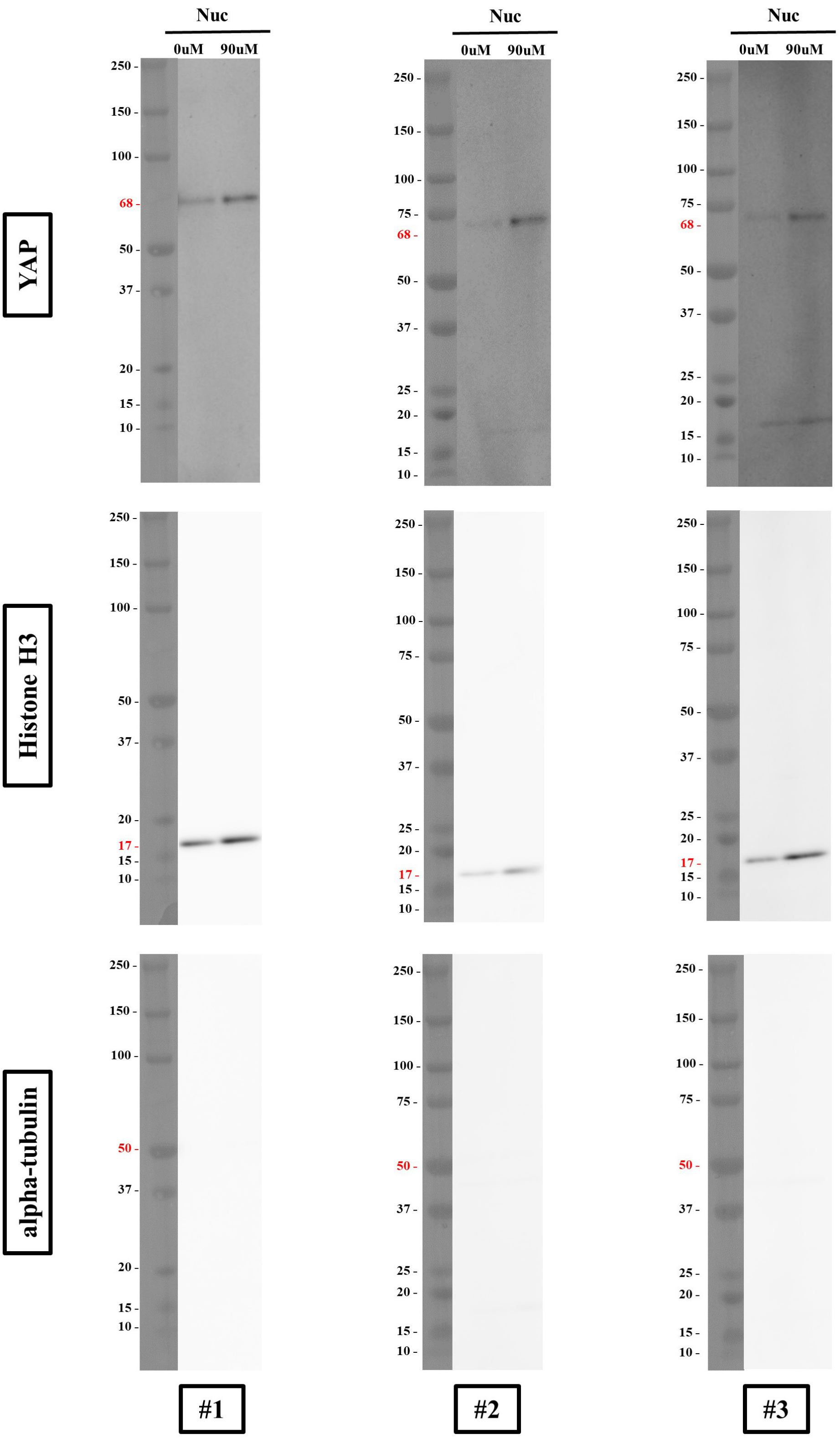

Figure S5. Western blot analysis of nuclear YAP in untreated- and DDT-Muse cells.

Images showing full membranes used for western blot analysis represented in figure 7C.

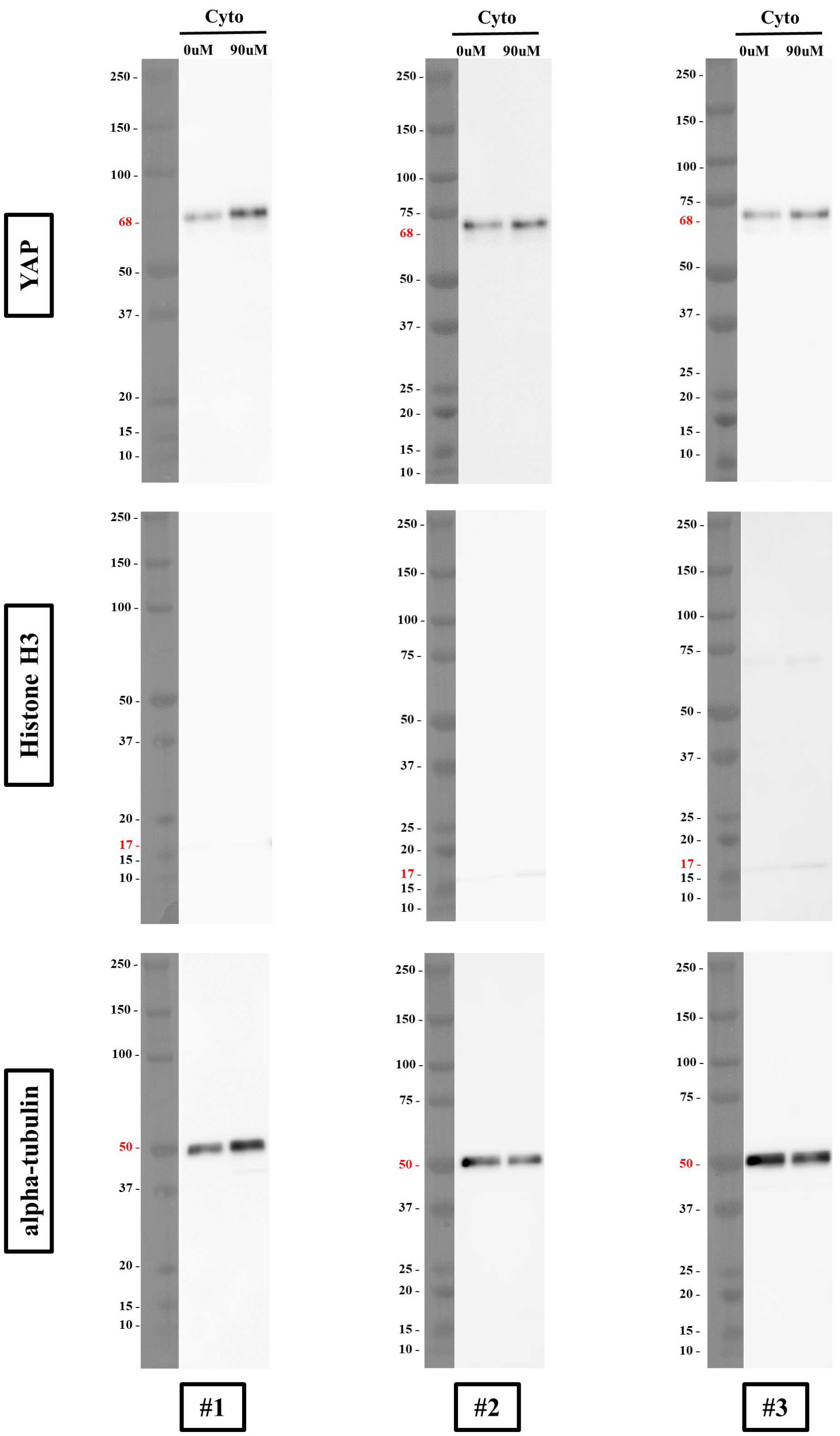

Figure S6. Western blot analysis of cytoplasmic YAP in untreated- and DDT-Muse cells.

Images showing full membranes used for western blot analysis represented in figure 7C.
